# Reliability, repeatability, and accordance between three different corneal diagnostic imaging devices for evaluating the ocular surface

**DOI:** 10.3389/fmed.2022.893688

**Published:** 2022-07-29

**Authors:** Abril L. Garcia-Terraza, David Jimenez-Collado, Francisco Sanchez-Sanoja, José Y. Arteaga-Rivera, Norma Morales Flores, Sofía Pérez-Solórzano, Yonathan Garfias, Enrique O. Graue-Hernández, Alejandro Navas

**Affiliations:** ^1^Department of Cornea and Refractive Surgery, Conde de Valenciana Institute of Ophthalmology, Mexico City, Mexico; ^2^Faculty of Medicine, Autonomous University of Baja California, Mexicali, Baja California, Mexico; ^3^School of Medicine, Panamerican University, Mexico City, Mexico; ^4^Faculty of Health Sciences North Campus, Anáhuac University, Mexico City, Mexico

**Keywords:** dry eye disease, diagnostic imaging, ocular surface, topography, diagnosis

## Abstract

**Purpose:**

To evaluate repeatability, reproducibility, and accordance between ocular surface measurements within three different imaging devices.

**Methods:**

We performed an observational study on 66 healthy eyes. Tear meniscus height, non-invasive tear break-up time (NITBUT) and meibography were measured using three corneal imaging devices: Keratograph 5M (Oculus, Wetzlar, Germany), Antares (Lumenis, Sidney, Australia), and LacryDiag (Quantel Medical, Cournon d’Auvergne, France). One-way ANOVAs with *post hoc* analyses were used to calculate accordance between the tear meniscus and NITBUT. Reproducibility was assessed through coefficients of variation and repeatability with intraclass correlation coefficients (ICC). Reliability of meibography classification was analyzed by calculating Fleiss’ Kappa Index and presented in Venn diagrams.

**Results:**

Coefficients of variation were high and differed greatly depending on the device and measurement. ICCs showed moderate reliability of NITBUT and tear meniscus height measurements. We observed discordance between measurements of tear meniscus height between the three devices, F2, 195 = 15.24, *p* < 0.01. Measurements performed with Antares were higher; 0.365 ± 0.0851, than those with Keratograph 5M and LacryDiag; 0.293 ± 0.0790 and 0.306 ± 0.0731. NITBUT also showed discordance between devices, F2, 111 = 13.152, *p* < 0.01. Measurements performed with LacryDiag were lower (10.4 ± 1.82) compared to those of Keratograph 5M (12.6 ± 4.01) and Antares (12.6 ± 4.21). Fleiss’ Kappa showed a value of -0.00487 for upper lid and 0.128 for inferior lid Meibography classification, suggesting discrete to poor agreement between measurements.

**Conclusion:**

Depending on the device used and parameter analyzed, measurements varied between each other, showing a difference in image processing.

## Introduction

An increasing number of adults worldwide experience dry eye symptoms, with a global prevalence of about 15% (1). This number seems to get higher with age, with an increasing prevalence among adults greater than 50 years of age (2).

Diagnosis and monitoring of dry eye disease (DED) may be achieved through the use of subjective scales such as the ocular surface disease index (3) and clinical exam findings such as tear breakup time, tear meniscus height, meibography, and interferometry in order to assess Meibomian gland dysfunction, among other causes of DED (4).

Meibomian glands are a variant of sebaceous glands that are at the tarsal plates of the superior and inferior eyelids. Each gland is composed of multiple secretory acini, lateral ducts, central conduct, and a terminal excretory conduct that converge at the eyelid posterior margin. The dysfunction of these glands is the most common identifiable cause of dry eye, with a prevalence of up to 41.7% (5). Meibography allows us to make a non-invasive, *in vivo* evaluation of Meibomian glands (6), where the morphology, architecture and percentage loss may be analyzed (7), and be vital for Meibomian gland dysfunction diagnosis.

Dry eye disease is an entity that affects both the tear film and the ocular surface. The tear meniscus is a tiny strip of tear fluid at the upper and lower lid margins and is therefore considered an important measurement in DED diagnosis (8), as it exemplifies loss of eye lubrication, and its measurement correlates well with the objective signs and subjective symptoms presented by DED. Non-invasive tear break-up time (NITBUT) is the time taken in seconds between the last blink and the first random disturbance of a grid on the corneal surface. It represents another easy to apply, non-invasive and fast method of evaluating tear function (9), as lower tear break up times are associated with DED.

The Keratograph 5M (Oculus, Wetzlar, Germany), Antares (Lumenis, Sidney, Australia), and LacryDiag (Quantel Medical, Cournon d’Auvergne, France) devices are novel corneal topographer devices developed to be used as an auxiliary diagnostics and follow-up tool. They all contain non-invasive functions to analyze various ocular surface measurements with acceptable sensitivity and specificity (10–12).

The purpose of this study is to evaluate repeatability, reliability, and accordance between ocular surface measurements among three different corneal diagnostics imaging devices.

## Materials and methods

This is an observational study on healthy subjects without any systemic or ocular disease, nor previous refractive surgery. Subjects who regularly used contact lenses were excluded. Participants were recruited during a 6-month period. This study was approved by our Institutional Review Board. The volunteers were informed about the purpose of the study and all work presented adheres to the Declaration of Helsinki.

**FIGURE 1:**
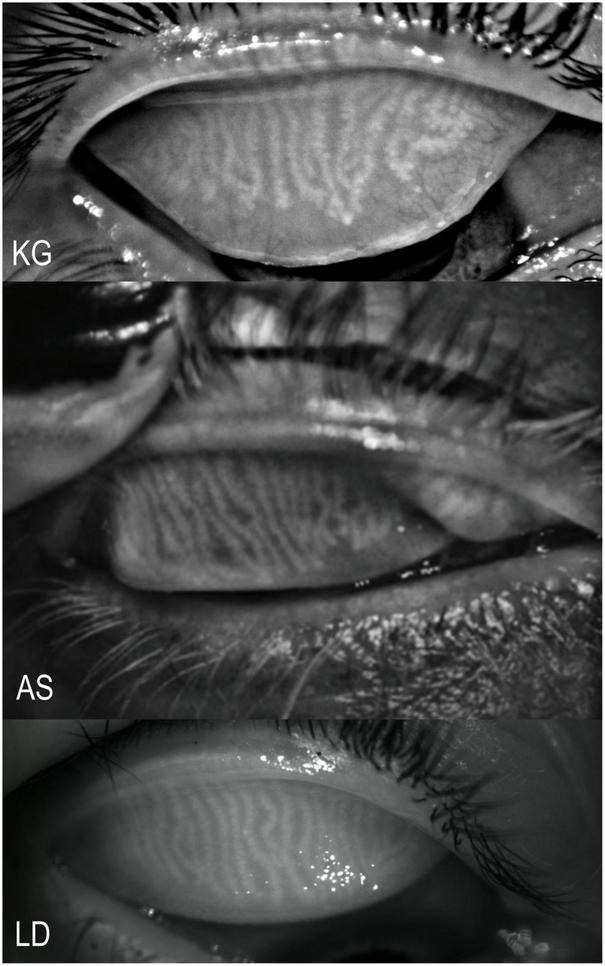
Examples of meibography imaging by the three devices. KG, Keratograph 5M; AS, Antares; and LD, LacryDiag.

We performed tear meniscus height measurement, NITBUT and meibography using three types of corneal imaging devices: Keratograph 5M, LacryDiag, and Antares. Studies were performed in this device order with a 5-min time interval between them. Examples of the meibography imaging performed by these devices are shown in Figure 1.

Meibography imaging was then classified according to the percentage of lost area, as described by the topographers: Degree 0 (0%), Degree 1 (<33%), Degree 2 (33–67%), and Degree 3 (>67%). Interferometry was only measured with Keratograph 5M and LacryDiag since in Antares this measure was not available. The evaluation was performed on both eyes in the same day to all participants by a well-trained examiner.

During the execution of the exams for all three devices, the volunteers were asked to place their chin and forehead on the device supports. The subjects were then asked to keep their eyes open and to fixate on a blinking target. The patient’s eyes were visualized on a computer screen, a joystick was used to take the scans and measurements. For tear meniscus height measurement, a caliper was used to assess where the tear meniscus begins and ends, to show a final height determined by the user. For NITBUT, the patient’s eye was recorded until they blinked, or until the device determined that the time for tear break up had been reached, with the analysis showing both first tear break up time and mean tear break up time. For meibography imaging, the patients’ eyelids were everted to expose the Meibomian glands, afterward the devices determined the percentage of loss using different techniques. Finally, we proceeded to compare the measurements obtained with all three devices.

### Statistical analysis

Sample size was determined using GPower 3.1 software computing a difference between three independent means. We set α at 0.05; 1-β at 0.95 and effect size at 0.4, calculating for a minimum of 0.8 power a total sample size of 62. All data was entered into R version 4.0.2 (13), where mean, standard deviation, maximum and minimum values for each parameter set was calculated. One-way ANOVA’s and Welch’s ANOVA’s (14) were used to calculate the level of statistical significance and the correlation between the three imaging devices. Reproducibility was assessed through coefficients of variation. Repeatability was analyzed between the left and right eyes of the subjects to search for similarity with intraclass correlation coefficients (ICC) (15). Reliability and accordance from the tear meniscus and NITBUT analyses were defined using Tukey Honest Significant Differences as well as Bonferroni corrections and plotted in Tukey mean difference plots with 95% confidence interval of limits of agreement. *P* values less than 0.05 were considered statistically significant (16). The accordance based on the meibography classification was analyzed by calculating Fleiss’ Kappa Index. Interpretation of this index was performed based on the classification proposed by Landis and Koch (17). Visualization of this agreement is presented visually in a logical (Venn) diagram to show the overlap in the classification performed by the three devices.

## Results

We studied 66 eyes of 33 individuals with a mean age of 27.2 ± 6.1 years (range 20–41), with a majority of female subjects (*n* = 20). The main data collected with all three devices are summarized in Table 1 for tear meniscus height, and Table 2 for NITBUT.

**TABLE 1:** Mean, standard deviation (SD), maximum, and minimum values collected by all three devices for tear meniscus height measurements.

	Tear meniscus (mm)			
**Device**	**Mean**	**SD**	**Max**	**Min**

Keratograph 5M	0.293	±0.0790	0.53	0.13
Antares	0.365	±0.0851	0.71	0.21
LacryDiag	0.306	±0.0731	0.56	0.18

**TABLE 2:** Mean, standard deviation (SD), maximum, and minimum values collected by all three devices for non-invasive tear break-up time measurements.

	NITBUT (seconds)			
**Device**	**Mean**	**SD**	**Max**	**Min**

Keratograph 5M	12.6	±4.01	22.2	5.61
Antares	12.6	±4.21	17.9	4
LacryDiag	10.4	±1.82	15.8	6.2

Coefficients of variation showed higher reproducibility with LacryDiag (CV = 0.17), compared to Keratograph 5M (CV = 0.31) and Antares (CV = 0.33), when measuring NITBUT. On the other hand, when analyzing tear meniscus height, similar reproducibility was achieved with both Antares (CV = 0.23) and LacryDiag (CV = 0.23), compared to Keratograph 5M (CV = 0.26). ICC translated to moderate reliability when measuring both NITBUT (ICC = 0.585) and tear meniscus height (ICC = 0.547).

When analyzing the tear meniscus, we observed disagreement between the measurements of the three devices, F_2,195_ = 15.24, *p* < 0.01. Measurements performed with Antares were significantly higher; 0.365 mm ± 0.0851 mm, than those with both the Keratograph 5M and LacryDiag; 0.293 mm ± 0.0790 mm and 0.306 mm ± 0.0731, respectively. The *post hoc* Tukey test showed that both Keratograph and LacryDiag measurements differed significantly from Antares, at *p* < 0.01; differences between Keratograph 5M and LacryDiag measurements were not significantly important.

Non-invasive tear break-up time measurements also showed disagreement between devices, F_2,111_ = 13.152, *p* < 0.01. In this case, measurements performed with LacryDiag were significantly lower (10.4 s ± 1.82 s) compared to those obtained with Keratograph 5M (12.6 s ± 4.01 s) and Antares (12.6 s ± 4.21 s). *Post hoc* analysis showed significant differences in the measurements performed by LacryDiag in comparison with the other two devices, at *p* < 0.01. Differences between measurements with Keratograph 5M and Antares were not statistically significant.

To review the accordance between Meibography classification with the three devices, a Fleiss’ Kappa coefficient was determined, showing a value of –0.00487 for the upper lid and 0.128 for the inferior lid. We also determined the Kappa coefficient for both the upper and lower lid with a value of 0.019. All three of these values suggest discrete to poor agreement between the measurements. We performed the same analysis pairing the devices. When comparing agreement between LacryDiag and Keratograph 5M on the upper lid, the value obtained was 0.0468, between LacryDiag and Antares the value was –0.0495, and between Keratograph 5M and Antares, –0.0443. On the other hand, when comparing the accordance between LacryDiag and Keratograph 5M on the lower lid, the value was 0.0767, between LacryDiag and Antares, the value found was 0.254, and between Keratograph 5M and Antares, 0.0819.

**FIGURE 2:**
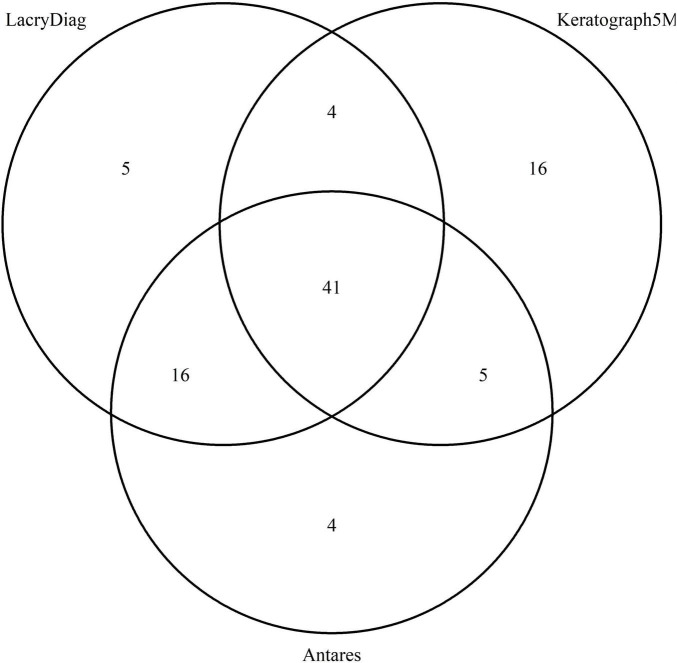
Venn diagram showing the overlap in upper lid classification of meibography performed by Keratograph 5M, Antares, and LacryDiag devices.

When we analyze the agreement in the logical diagram, 41 (62.12%) of upper lid meibography images were categorized in the same degree by all three devices, 4 (6.06%) were equally categorized by LacryDiag and Keratograph 5M, 16 (24.24%) by LacryDiag and Antares, and 5 (7.57%) were classified the same in both Keratograph 5M and Antares. This is shown in Figure 2.

**FIGURE 3:**
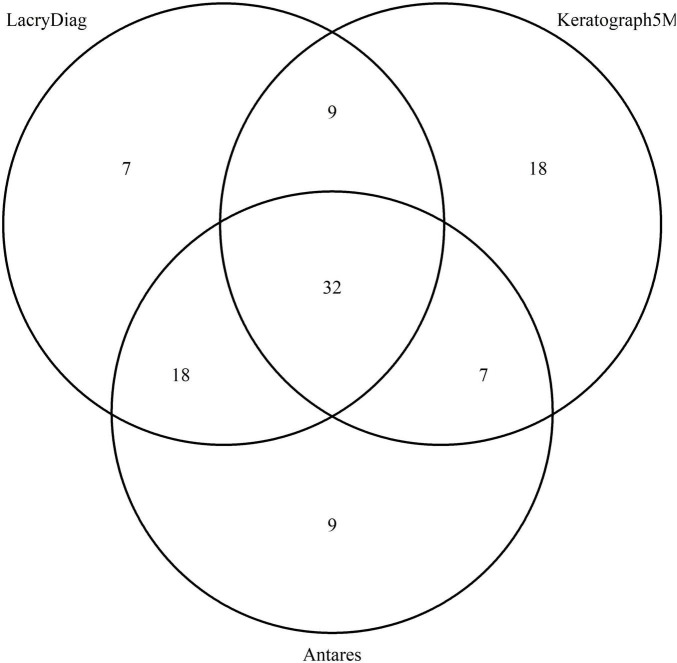
Venn diagram showing the overlap in lower lid classification of meibography performed by Keratograph 5M, Antares, and LacryDiag devices.

Lower lid images showed a similar distribution: 32 (48.48%) were classified in the same degree by the three devices, 9 (13.63%) when reviewing LacryDiag versus Keratograph 5M, 18 (27.27%) overlapped between LacryDiag and Antares, and 7 (10.60%) were classified the same in both Keratograph 5M and Antares, as presented in Figure 3.

Interferometry was measured quantitatively by the LacryDiag device, unlike Keratograph 5M which makes a qualitative analysis. Antares did not count with an interferometry analysis. Due to this, we were unable to compare the measurements performed by the devices.

## Discussion

Dry eye disease is of increasing concern due to a high prevalence, even higher expenses and economic burden for both individuals and health systems, making adequate identification and diagnosis extremely important (18). The advent of new technologies to evaluate the ocular surface has allowed the analysis of corneal diseases with augmented ease. The development of specialized imaging devices, such as ocular surface topographers allows for non-invasive evaluation of various measurements (19). However, it is important to know if these measurements are interchangeable so as to be able to utilize these values for the diagnosis and management of eye diseases, no matter the device used.

Previous studies have been performed to compare different devices for evaluating DED (20), with good enough repeatability and reliability. However, agreeability between these many devices has not ever been performed before. Even more, the increasing number of new devices available in the market make for a difficult choice in deciding where to invest. Our study shows that depending on the parameter analyzed, different devices might show agreeability, while others do not.

Regarding reproducibility, our coefficients of variation were high, with different results depending on the device and measurement analyzed. We infer that these percentages of variability are due to the high sensitivity of the devices, more than to unreliable results. On the other hand, ICC show moderate reliability of the measurements performed which may again account for the sensitivity of these devices.

When analyzing the tear meniscus, previous studies show that these specialized imaging devices are able to perform accurate measurements (21). Our results demonstrate that Antares measures this parameter differently than the other two devices. Antares appears to overestimate the length of tear meniscus height, potentially underestimating dry eye diagnosis. This may be due to differences in the accuracy of the caliper of each device’s software when measuring the length of the meniscus, as it depends greatly on the user and maybe even to the computer mouse’s sensitivity.

On the other hand, NITBUT measurements showed different values reported by LacryDiag, demonstrating a shorter time when compared to those performed by the other two devices. Our measurements with the three devices were performed sequentially on the same day, using the LacryDiag device at the end, which may have caused the variation between the values obtained. However, the high level of agreeability between the other two devices might suggest that the reason for this difference might rely more on a higher sensitivity of LacryDiag image processing, and thus underestimate tear breakup time.

Finally, when comparing meibography images, slight differences were discovered between all three devices, being more pronounced between Keratograph 5M and the other two devices. This may well be because of the noticeable differences in the way these images were processed. Keratograph 5M allows the evaluator to classify the image in four stages according to the amount of meibomian gland loss, therefore making the assessment considerably subjective. Antares allows for the evaluator to create an estimated area of analysis, consecutively discerning the approximate area of loss. LacryDiag performs a similar analysis where the user must highlight an approximate area where meibomian glands are present and subsequent analysis is performed based on what the user pointed out. Furthermore, in all three devices, the image is taken, and the analysis is performed through different LED infrared diodes, being 875 for Antares and 840 for Keratograph 5M. LacryDiag does not specify the diode wavelength, but we presume it could be different because of the results obtained. The contrast of Meibomian gland images has been measured before. In a previous study, ten subjects were evaluated with a range of wavelengths varying from 600 to 1,050 nm. The authors found different values of contrast when Meibomian glands are illuminated at different wavelengths. We believe this could also account for the diverse results portrayed (22). On the other hand, despite the different ways of determining the percentage of meibomian gland loss between the devices, the majority of images were still classified within the same degree, as shown by the Venn diagrams, suggesting that these differences might not affect the clinical assessment of patients.

Due to the differences accounted in the study, we recommend that physicians should consider using the device they feel more comfortable with, whichever they consider having the easiest user interface, or the one that seems more comfortable for the patient, rather than aiming for complete interchangeability. In this same tenant, we recommend for physicians to use the same machine for diagnosis and follow up of patients. However, ease of use and comfortability were not parameters studied and were not the aim of this research.

In conclusion, measurements performed by the different devices analyzed in this study vary between each other, possibly reflecting differences in image processing. Depending on the image to be analyzed, specialized imaging devices might show varying results.

## Data availability statement

The raw data supporting the conclusions of this article will be made available by the authors, without undue reservation.

## Ethics statement

The studies involving human participants were reviewed and approved by Instituto de Oftalmología “Conde de Valenciana” Ethics Committee. The patients/participants provided their written informed consent to participate in this study.

## Author contributions

AG-T and FS-S collected, analyzed, and interpreted all data collected, drafted, and revised the manuscript. DJ-C designed the statistical design plan, analyzed, and interpreted the data collected, drafted, and analyzed the manuscript. JA-R drafted and revised the manuscript. NM and SP-S conceptualized and designed the work and monitored data collection. EG-H and AN revised the work and gave final approval for publication. All authors contributed to the article and approved the submitted version.
